# Successful treatment for disseminated intravascular coagulation (DIC) corresponding to phenotype changes in a heat stroke patient

**DOI:** 10.1186/s40560-019-0359-3

**Published:** 2019-01-15

**Authors:** Hironori Matsumoto, Jun Takeba, Kensuke Umakoshi, Yuki Nakabayashi, Naoki Moriyama, Suguru Annen, Muneaki Ohshita, Satoshi Kikuchi, Norio Sato, Mayuki Aibiki

**Affiliations:** Graduate School of Medicine, Department of Emergency and Critical Care Medicine, Ehime University, Shitsukawa 454, Toon, Ehime 791-0295 Japan

**Keywords:** Anticoagulant, Antithrombin, Disseminated intravascular coagulation, Heat stroke, Soluble fibrin, Thrombomodulin, Thrombin-antithrombin complex

## Abstract

**Background:**

Heat stroke induces coagulofibrinolytic activation, which leads to life-threatening disseminated intravascular coagulation (DIC). However, treatment strategies for DIC in heat stroke have not yet been established, and also, the time course changes in coagulofibrinolytic markers have not been thoroughly evaluated. We report a severe heat stroke case with DIC who was eventually saved by anti-DIC treatments in accordance with changes in coagulofibrinolytic markers.

**Case presentation:**

A 45-year-old man was found unconscious outside, and his body temperature was elevated to 41.9 °C. For heat stroke, we performed an immediate tracheal intubation under the general anesthesia along with cooling by iced gastric lavage, cold fluid administration, and an intravascular cooling using Thermogard™. About 4 h after admission, his core temperature fell to 37 °C. We assessed coagulofibrinolytic biomarkers and treated in accordance with changes in these parameters. This case exhibited a biphasic change varying from an enhanced to a suppressed fibrinolytic type of DIC depending on the relative balance between fibrinolytic activation and the level of plasminogen activator inhibitor-1 (PAI-1). In the early phase with consumption coagulopathy and enhanced fibrinolysis, we transfused a large amount of fresh frozen plasma (FFP) and platelets with tranexamic acid, an antifibrinolytic agent, possibly providing relief for the bleeding tendency. Anticoagulant therapy using recombinant human thrombomodulin-α (rh-TM-α) and antithrombin III (ATIII) concentrate was especially effective for DIC with a suppressed fibrinolytic phenotype in the later phase, after which organ failure that included severe hepatic failure was remarkably improved.

**Conclusion:**

The present case may indicate the clinical significance of monitoring coagulifibrinolytic changes and the potential benefits of anticoagulants for heat stroke-induced DIC.

## Background

Heat stroke often causes hypercoagulation simultaneously with enhanced fibrinolysis [1, 2], leading to disseminated intravascular coagulation (DIC) with a grave outcome [2–4]. The treatment strategy for DIC in heat stroke, however, has not been established, and the time course of DIC in heat stroke has not been thoroughly evaluated. We report here a full recovery case treating in accordance with changes in coagulofibrinolytic parameters. The time course of heat stroke in the current case showed a drastic DIC phenotype changing from an enhanced to a suppressed fibrinolytic phenotype depending on the balance between the initial coagulofibrinolytic activation and the PAI-1 elevation. In the early phase with enhanced-fibrinolytic DIC, we transfused a large amount of FFP and platelets with an antifibrinolytic agent, which eventually resulted in the relieving and the bleeding tendency. To treat the hypercoagulation with suppressed fibrinolysis resulting from an elevation of plasminogen activator inhibitor-1 (PAI-1) that occurred in the later phase, we performed a combination therapy using recombinant human thrombomodulin-α (rh-TM-α) with antithrombin III (ATIII) concentrate, after which DIC was remarkably resolved.

## Case presentation

On a hot spring day, a 45-year-old man was found unconscious outside of his house after having spent about 5 h mowing grass. On admission, the patient was in a deep coma, his axillary body temperature was elevated to 41.9 °C (42.3 °C in the ambulance), and his respiratory and heart rates rose to 30 and 176 bpm, respectively. As initial treatments for this apparent heat stroke patient, we performed an immediate tracheal intubation under general anesthesia along with cooling by iced gastric lavage, cold fluid administration, and an intravascular cooling using Thermogard™. About 4 h after admission, his core temperature fell to 37 °C.

**Fig. 1:**
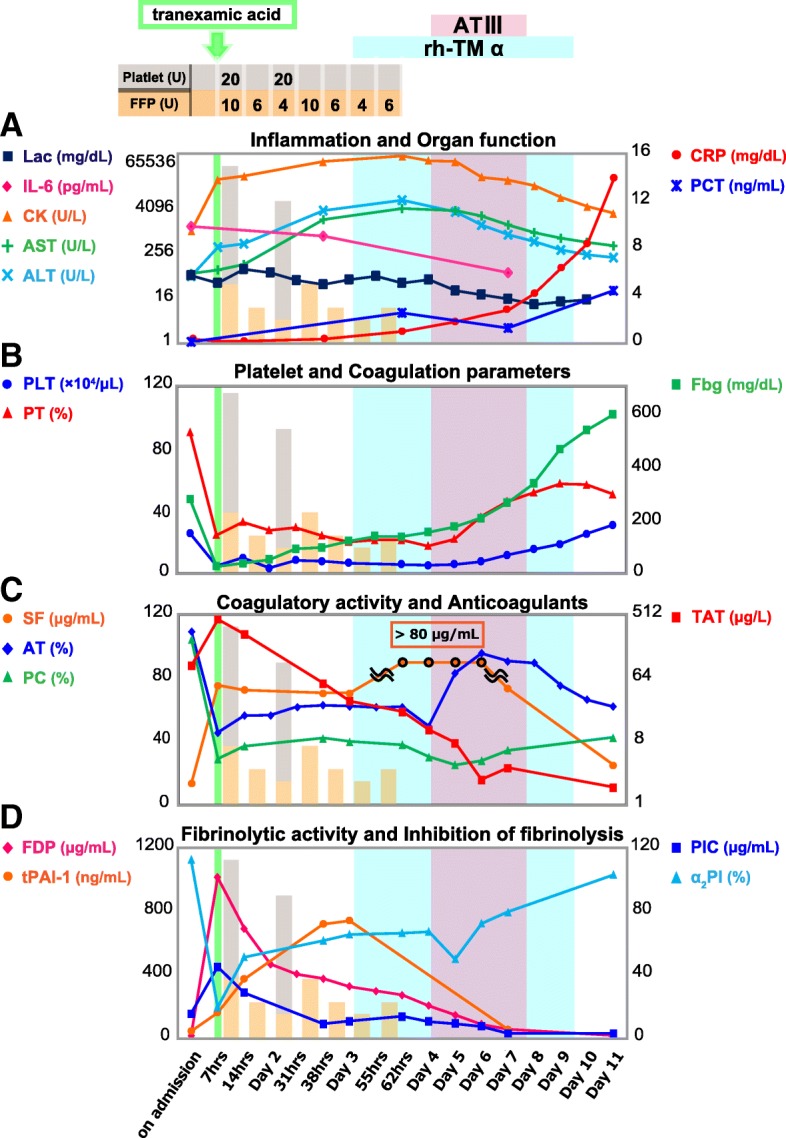
Time course of laboratory parameters and treatments. Panels **a** and **b** show changes in bio-chemical parameters and coagulation factors. Note that after the supplementation of ATIII concentrate, AST and ALT (**a**) remarkably decreased with associations of PT and PLT (**b**) improvements. CRP (**a**) did not respond despite high IL-6 levels (**a**), after ATIII concentrate, unexpectedly increased, which might show a possible improvement in hepatic CRP synthesis. Panels **c** and **d** demonstrate prominent elevations in TAT (**c**), FDP and PIC (**d**) in the early phase, which was accompanied with a steep drop in α_2_PI (**d**). tPAI-1 (**d**) increased gradually, peaking on day 3, which was followed by a sustained SF (**c**) soaring. Although TAT dropped gradually, SF augmentation was not attenuated. After the combined administration of rh-TM-α and ATIII concentrate, further decrease in TAT with a gradual fall in SF occurred, along with changes in bio-chemical parameters stated above

For more than 10 years, he had been taking antipsychotics to treat his schizophrenia, but his disease state had been stable and no changes had been made to the drugs or their dosages. Furthermore, there were no signs of muscle stiffness suggesting neuroleptic malignant syndrome. His procalcitonin level was low (0.087 ng/mL, Fig. 1a), and the bacteriological examinations showed no evident infection. Other examinations, including whole body computed tomography, also showed no findings suggesting other causes for the elevated fever.

The blood examination on admission showed increases in coagulofibrinolytic activity (thrombin-antithrombin complex [TAT] 97.1 μg/L, soluble fibrin [SF] 13.6 μg/mL, plasmin-α_2_-plasmin inhibitor complex [PIC] 15.3 μg/mL, fibrin/fibrinogen degradation products [FDP] 14.3 μg/mL; panels c and d) without bleeding tendency. Total plasminogen activator inhibitor-1 (tPAI-1), a bio-substance inhibiting fibrinolysis, did not increase on admission (43 ng/mL, panel d).

About 7 h after admission, gastrointestinal hemorrhage and oozing from catheter puncture sites occurred. The coagulofibrinolytic markers at that time point showed remarkable further increases (TAT 443.5 μg/L, SF 75.6 μg/mL, PIC 44.9 μg/mL, FDP 1014.0 μg/mL; panels c and d) with consumption coagulopathy (platelet [PLT] 4.6 × 10^4^/μL, prothrombin time [PT] 24.5%, fibrinogen [Fbg] 25 mg/dL, α_2_-plasmin inhibitor [α_2_PI] 19.5%; panels b and d), eventually complicating with enhanced-fibrinolytic DIC (DIC score: International Society on Thrombosis and Hemostasis [ISTH], 7; Japanese Association for Acute Medicine [JAAM], 8). Diagnosis of the DIC phenotype was made in accordance with Asakura’s criteria [5]. At this time, tPAI-1 elevation was still relatively mild compared with the coagulofibrinolytic activation (160 ng/mL; panel d). This DIC was complicated by reductions in anticoagulatory factors (AT 45.7%, protein C [PC] 29.3%; panel c). We administered 1000 mg of tranexamic acid and transfused fresh frozen plasma (FFP) with concentrated platelets (upper portion of the Fig. 1). Thereafter, the bleeding tendency gradually improved, and the activated coagulofibrinolysis peaked. Still, the consumption coagulopathy was sustained, so continued blood transfusion was needed. Also, liver damage reflected by AST and ALT increases was worsening (panel a).

On day 3, the reductions in anticoagulants (AT 63.0%, PC 42.4%; panel c) continued with sustained hypercoagulation (TAT 54.1 μg/L, SF 70.7 μg/mL; panel c), which could possibly have prolonged the consumption coagulopathy (PLT 7.7 × 10^4^/μL, PT 24.0%; panel b). tPAI-1 gradually increased to the peak level of 720 ng/mL (panel d) with decreasing fibrinolytic activity on the same day (PIC 8.8 μg/mL, FDP 373.4 μg/mL; panel d). Thus, these alterations indicated a change to a suppressed-fibrinolytic type of DIC (DIC score: ISTH, 6; JAAM, 8). Therefore, we administered rh-TM-α (130 U/kg/day) for the purpose of anticoagulation from day 3 (upper portion of Fig. 1). On day 4, SF still increased beyond the highest measurable level of 80 μg/mL (panel c), reflecting a continuation of the hypercoagulatory state. That change was followed by an abrupt AT decrease to 49.7% (panel c). We therefore added ATIII concentrate at an initial loading of 1500 U for 3 h followed by a continuous infusion of 1500 U/day for 3 days (upper portion of Fig. 1). This combined anticoagulation therapy improved not only the hypercoagulation but also the consumption coagulopathy, resulting in the resolution of DIC (DIC score: ISTH, 0; JAAM, 1). In the amelioration of hepatic dysfunction showing as decreases in highly elevated AST and ALT, there was a particularly dramatic recovery in PT values (panels a and b). After these catastrophic episodes, the patient eventually regained consciousness and was transferred to another hospital for further rehabilitation.

## Discussion

### The changes in fibrinolytic activity associated with plasma PAI-1 levels in DIC

PAI-1 plays an important role in the regulation of fibrinolysis. This antifibrinolytic substance exerts a fast-acting inactivation of released tissue-type plasminogen (t-PA) by forming a stoichiometric 1:1 complex (tissue-type plasminogen activator-plasminogen activator inhibitor complex [tPAIC]). PAI-1 exists only a small amount in the circulating blood under normal conditions (< 40 ng/mL) and is secreted from endothelial cells in response to inflammatory reactions [6]. The expression of PAI-1 mRNA occurs, after the immediate t-PA release, however, causing an imbalance of these two molecules. This time difference between t-PA and PAI-1 responses could cause hyperfibrinolysis [7].

In heat stroke, systemic inflammation and thermal injury to vascular endothelia leads to a coagulofibrinolytic activation including t-PA release [1, 2, 8]. Excessively released t-PA immediately after heat exposure in the absence of a concomitant increase of PAI-1 causes hyperfibrinolysis, which is subsequently suppressed by a gradual elevation in PAI-1 [3, 9]. In the early phase of the current case, hyperfibrinolysis represented by PIC elevation without a concomitant increase in tPAI-1 was found when the bleeding tendency developed. tPAI-1 represents a total concentration of active PAI-1 antigen and tPAIC. In the early phase of the course, it is likely that PAI-1 antigen might stay low, possibly due to its delayed mRNA expression. Furthermore, this low PAI-1 antigen could form only a minimal amount of tPAIC despite the increased t-PA release, resulting in low tPAI-1 levels. These hyperfibrinolytic conditions could enhance massive plasmin generation and consume α_2_PI, leading to enhanced-fibrinolytic DIC. On the contrary in the later phase, tPAI-1 gradually increases enough to inactivate increased t-PA, and thereafter, the fibrinolytic activation becomes suppressed.

We also found that, in this case, initial coagulation and fibrinolytic activation continued to increase even after the normalization of the body temperature; thereafter, the fibrinolytic inhibition and the improvement of the bleeding tendency occurred in parallel with tPAI-1 elevation. In contrast to the findings of this case, the fibrinolytic inhibition after heat stroke has been thought to result from normalization of the body temperature [1, 10]. However, as shown in this report, changes in fibrinolytic activity may depend on a relative balance between fibrinolytic activation and increasing PAI-1 levels. Thus, we need to consider changes in fibrinolytic activity associated with PAI-1 alterations even after the normalization of body temperature.

### Anti-DIC treatment corresponding to changes of DIC phenotypes in heat stroke

In the early phase of the current case with consumption coagulopathy and hyperfibrinolysis, we diagnosed the patient with enhanced-fibrinolytic DIC based on Asakura’s criteria [5]. In accordance with the DIC treatment guidelines [11, 12], we transfused a large amount of FFP and platelets with tranexamic acid, an antifibrinolytic agent, possibly providing relief of the bleeding tendency. As for the antifibrinolytic agent, we administered 1000 mg of tranexamic acid in accordance with the CRASH-2 trial, which was a large-scale randomized controlled trial investigating the effects of the agent on mortality, vascular occlusive events, and transfusion requirement in bleeding trauma patients [13], since in terms of the coagulofibrinolytic responses, this case exhibited similar changes to those in the early phase of trauma [14]. We did not perform a subsequent continuous infusion of tranexamic acid, considering the gradual tPAI-1 increase and sustained hypercoagulation, which could involve a potential risk of thrombin formation. In the future, the optimal method and efficacy of tranexamic acid for enhanced-fibrinolytic DIC in heat stoke need to be clarified.

In the later phase of this case with suppressed fibrinolysis, prolonged hypercoagulation might have caused multiple organ dysfunction syndrome (MODS). In Japan, rh-TM-α and ATIII concentrate are available for the treatment of DIC since several clinical studies have reported their efficacy in different types of DIC [15–17]. Also, in animal models of heat stroke, the benefits of rh-TM-α or ATIII concentrate have been reported [18, 19]. In the current case, rh-TM-α at a dose of 130 U/kg/day, a reduced dose for acute kidney injury recommended at that time might not have suppressed hypercoagulation in the later phase of heat stroke, which was revealed by sustained SF elevation. We therefore supplemented with ATIII concentrate, after which SF decreased and PT remarkably improved along with a gradual increase in PLT. Thus, this combined therapy of anticoagulants inhibited hypercoagulation that occurred in the later phase of heat stroke, which led to the amelioration of MODS, including severe liver failure, through the improvement of DIC. We should have administered rh-TM-α at a dose of 380 U/kg/day, the recommended dose for DIC. The current case had just recovered from a bleeding tendency, however, and was complicated with severe renal dysfunction (GFR < 10 mL/min/1.73 m^2^ on day 3), which made us treat carefully with renal excretory rh-TM-α at a dose of 380 U/kg/day. We thus selected an initial dose of 130 U/kg/day to prevent bleeding complications. At that time, the registered administration dose of rh-TM should be reduced depending on the patient’s renal function. Furthermore, after discontinuation of FFP transfusion, AT activity abruptly decreased, so we decided to add ATIII concentrate rather than an increase of the dosage of rh-TM-α. Although it remains unclear which method is appropriate, an anticoagulant therapy for a suppressed-fibrinolytic type of DIC in the later phase of heat stroke was very effective for the resolution of DIC in the current case. We need further studies to examine its efficacy and method.

DIC is classified by the degree of fibrinolytic activation: an enhanced type usually seen in the early phase of severe trauma, a suppressed type in sepsis, and a balanced type in solid tumor [5]. The appropriate diagnosis and treatment may differ depending on the DIC type [5, 11, 12]. An important point in the treatment of heat stroke is a careful monitoring of the phenotype changes in DIC from an enhanced to a suppressed fibrinolytic phenotype, depending on the balance between the initial coagulofibrinolytic activation and the PAI-1 elevation.

## Conclusion

The current case may shed light on an important point in the pathogenesis and treatment strategy of DIC in heat stroke: a possible time-dependent relationship between coagulofibrinolytic activation and PAI-1 changes. Therefore, as shown in this case, the treatment strategy for DIC corresponding to changes of phenotypes by PAI-1 changes could be a potential candidate.

## Abbreviations

AT: Antithrombin
DIC: Disseminated intravascular coagulation
Fbg: Fibrinogen
FDP: Fibrin/fibrinogen degradation products
FFP: Fresh frozen plasma
ISTH: International Society on Thrombosis and Hemostasis
JAAM: Japanese Association for Acute Medicine
MODS: Multiple organ dysfunction syndrome
PAI-1: Plasminogen activator inhibitor-1
PC: Protein C
PIC: Plasmin-α_2_-plasmin inhibitor complex
PLT: Platelet
PT: Prothrombin time
rh-TM-α: Recombinant human thrombomodulin-α
SF: Soluble fibrin
TAT: Thrombin-antithrombin complex
t-PA: Tissue-type plasminogen activator
tPAI-1: Total plasminogen activator inhibitor-1
α_2_PI: α_2_-plasmin inhibitor
